# External validation of a radiomic signature to predict p16 (HPV) status from standard CT images of anal cancer patients

**DOI:** 10.1038/s41598-023-34162-3

**Published:** 2023-05-03

**Authors:** Ralph T. H. Leijenaar, Sean Walsh, Akshayaa Vaidyanathan, Lorenzo Aliboni, Victoria Lopez Sanchez, Michelle Leech, Ronan Joyce, Charles Gillham, Frédéric Kridelka, Roland Hustinx, Denis Danthine, Mariaelena Occhipinti, Wim Vos, Julien Guiot, Philippe Lambin, Pierre Lovinfosse

**Affiliations:** 1 Radiomics (Oncoradiomics SA), Liège, Belgium; 2 The D-Lab, Department of Precision Medicine, GROW–School for Oncology and Reproduction, Maastricht University, Maastricht, The Netherlands; 3 Applied Radiation Therapy, Discipline of Radiation Therapy, Trinity St. James’s Cancer Institute, Trinity College, Dublin, Ireland; 4 Department of Radiation Oncology, St. Luke’s Radiation Oncology Network and St James’s Hospital, Dublin, Ireland; 5 Department of Obstetrics and Gynecology, University Hospital of Liège, Liège, Belgium; 6 Department of Nuclear Medicine and Oncological Imaging, University Hospital of Liège, Liège, Belgium; 7 Department of Pneumology, University Hospital of Liège, Liège, Belgium; 8 Department of Radiology and Nuclear Medicine, GROW–School for Oncology and Reproduction, Maastricht University, Maastricht, The Netherlands

**Keywords:** Cancer imaging, Gastrointestinal cancer

## Abstract

The paper deals with the evaluation of the performance of an existing and previously validated CT based radiomic signature, developed in oropharyngeal cancer to predict human papillomavirus (HPV) status, in the context of anal cancer. For the validation in anal cancer, a dataset of 59 patients coming from two different centers was collected. The primary endpoint was HPV status according to p16 immunohistochemistry. Predefined statistical tests were performed to evaluate the performance of the model. The AUC obtained here in anal cancer is 0.68 [95% CI (0.32–1.00)] with F1 score of 0.78. This signature is TRIPOD level 4 (57%) with an RQS of 61%. This study provides proof of concept that this radiomic signature has the potential to identify a clinically relevant molecular phenotype (i.e., the HPV-ness) across multiple cancers and demonstrates potential for this radiomic signature as a CT imaging biomarker of p16 status.

## Introduction

Human papillomavirus (HPV) is a common virus that can lead to certain types of cancer later in life. Several studies have shown that HPV status of cancer patients plays an important role in treatment outcomes^1,2^. In particular, HPV + patients with oropharyngeal cancer present a markedly better prognosis and improved response to conventional radiotherapy^3^. Also, patients with anal cancer show a better prognosis and response to therapy when HPV + ^4^. Widely accepted methods for detection of HPV infection are in situ hybridization for viral DNA, HPV DNA or RNA PCR, and immunohistochemical investigation of the level of p16 expression, which strongly correlates with HPV infection^1,5^. However, these methods are time consuming and require biological patient samples. Image data analysis represents a novel non-invasive method of delivering reliable and quality results from standard-of-care medical images. The extraction of clinically relevant information from medical imaging is called radiomics. Radiomics is a rapidly emerging field, introduced in 2012, which concerns with the high-throughput mining of large amounts of quantitative features, derived from medical image such as CT/MRI/PET scans^6,7^. Radiomics is promising within decision support systems for precision medicine and its potential to predict p16 status in head and neck cancer has been recognized^8^. Previous studies have reported radiologic differences between p16 positive and negative cases in terms of qualitative radiologist’s readout of perfusion CT^9^. Furthermore, exploratory radiomic studies have shown that heterogeneity of image-based density is potentially associated with p16 status in oropharyngeal squamous cell carcinoma (OPSCC)^10^. Most of the studies investigating imaging phenotypes of tumors are based on single center data, which introduce bias to a model and limits its applicability. In particular, factors such as CT scanner, tube voltage, tube current, reconstruction kernel and contrast agent influence the results of quantitative analyses^11^. In this independent external validation study, we further investigate if a quantitative CT-based radiomic approach can objectively identify the p16 status of anal cancer in addition to OPSCC^12^, by validating a radiomic signature on patient data from two different institutions. With this study we aim to provide a proof of concept for radiomics to derive molecular information from standard medical images. Moreover, we demonstrate that the already developed and validated radiomics signature for head and neck cancer can also be useful in identification of HPV status of anal cancer patients.

## Materials and methods

### Patient cohort

Two independent cohorts, for a total of 59 anal cancer patients treated with curative intent by surgery and/or radiation therapy with/without concurrent chemotherapy, were collected from University Hospital of Liège (CHU) (n = 18) and St James's Hospital (TCD). (n = 41) The patients cohort is composed of 12% of stage I, 40% of stage II, 45 of stage III and 3 of stage IV anal cancer. The HPV status of the patients was inferred from p16 immunohistochemistry status (54 + and 5−). All patients underwent pre-treatment CT imaging, according to the standard of care of the treating institution. The gross primary tumor volume (GTV) was manually segmented for each patient by experienced radiologists. Institutional review board approval was obtained from the Ethics committees of the University Hospital of Liege, Trinity College, Cork University Hospital and St. Luke Hospital. The need for informed consent was waived from the same ethics committees since the data were anonymized and retrospectively collected. The present work as been conducted in accordance with the Declaration of Helsinki.

### Radiomics analysis

**Figure 1:**
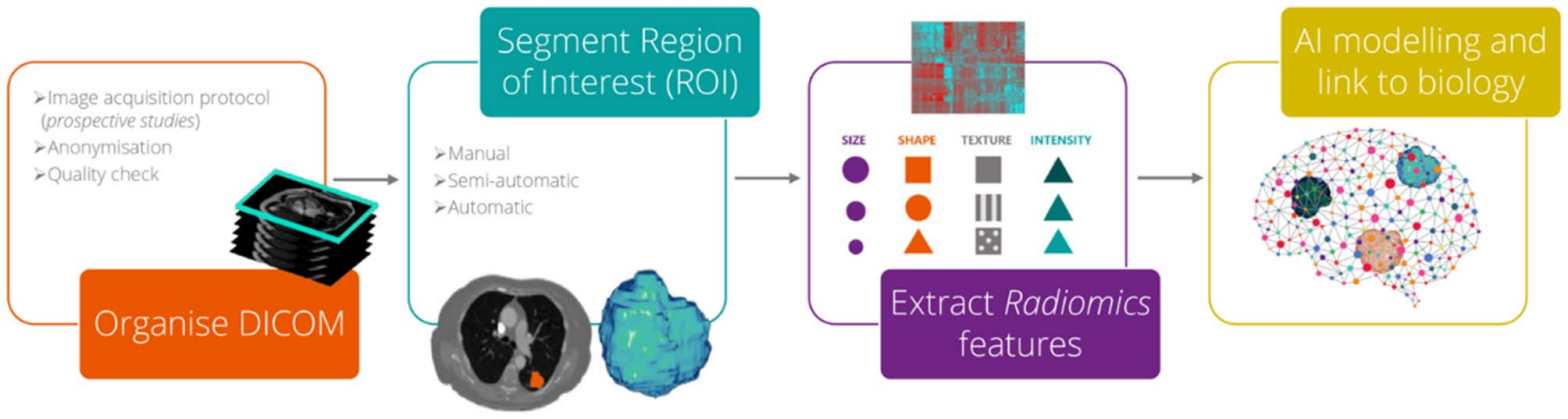
Radiomics workflow.

Radiomics is based on the hypothesis that quantitative analysis of medical image data via automatic or semi-automatic software can provide more and better information than that of a physician^13^. The schematic representation in Fig. 1 depicts the radiomics workflow applied in this study.

The radiomics workflow is divided in four mains steps. First the imaging data are collected, and eventually preprocessed, dividing them in different set (training validation and testing): then the region of interest (ROI) is segmented and annotated manually or (semi) automatically. From this region of interest handcrafted radiomics features are extracted, and divided into Size, Shape, Texture and Intensity features. The radiomics features are then used to train the AI model and the performances are validated in the test set and additionally in an external validation set.

In the external validation study presented here, prior to analysis, all images were resampled to isotropic voxels of 2 mm, using linear interpolation. A total of 37 radiomics features were calculated from five groups: tumor intensity, shape, texture, Wavelet and Laplacian of Gaussian. All features were extracted using the RadiomiX® software (OncoRadiomics SA, Liege, Belgium). Feature descriptions and mathematical definitions can be found in the literature^14^. Details for the development of the radiomics signature for HPV status in OPSCC are reported elsewhere^12^. Briefly, the signature was built using regularized logistic regression and showed an AUC of 0.763 [95% CI (0.687–0.839)] in development. The signature class predictions were made with a probability cut-off of 0.5.

### Statistical analysis

The signature performances have been evaluated in terms of Precision (1), Sensitivity (2), F1 score (3), Specificity and AUC.

1 $$\Pr ecision{\mkern 1mu} = {\mkern 1mu} True{\mkern 1mu} Positive{\mkern 1mu} value/(True{\mkern 1mu} Positive{\mkern 1mu} value + False{\mkern 1mu} Positive{\mkern 1mu} value)$$

2 $$Sensitivity = True{\mkern 1mu} Positive{\mkern 1mu} value/(True{\mkern 1mu} Positive{\mkern 1mu} value{\mkern 1mu} + {\mkern 1mu} False{\mkern 1mu} Negative{\mkern 1mu} value)$$

3 $$F1{\mkern 1mu} Score = 2{\mkern 1mu} *{\mkern 1mu} (\Pr ecision{\mkern 1mu} *{\mkern 1mu} Sensitivity)/(\Pr ecision{\mkern 1mu} + {\mkern 1mu} Sensitivity)$$

Assuming a 85% prevalence of HPV positive anal cancer patients^15^, we calculated also Negative predictive value (NPV) and Positive predictive value (PPV). The signature was also evaluated according to the Transparent Reporting of a multivariable prediction model for Individual Prognosis or Diagnosis (TRIPOD)^16^ and the Radiomics Quality Score (RQS)^14^. Statistical analysis was performed in R (R Foundation for Statistical Computing; v. 3.3.3).

## Results

**Figure 2:**
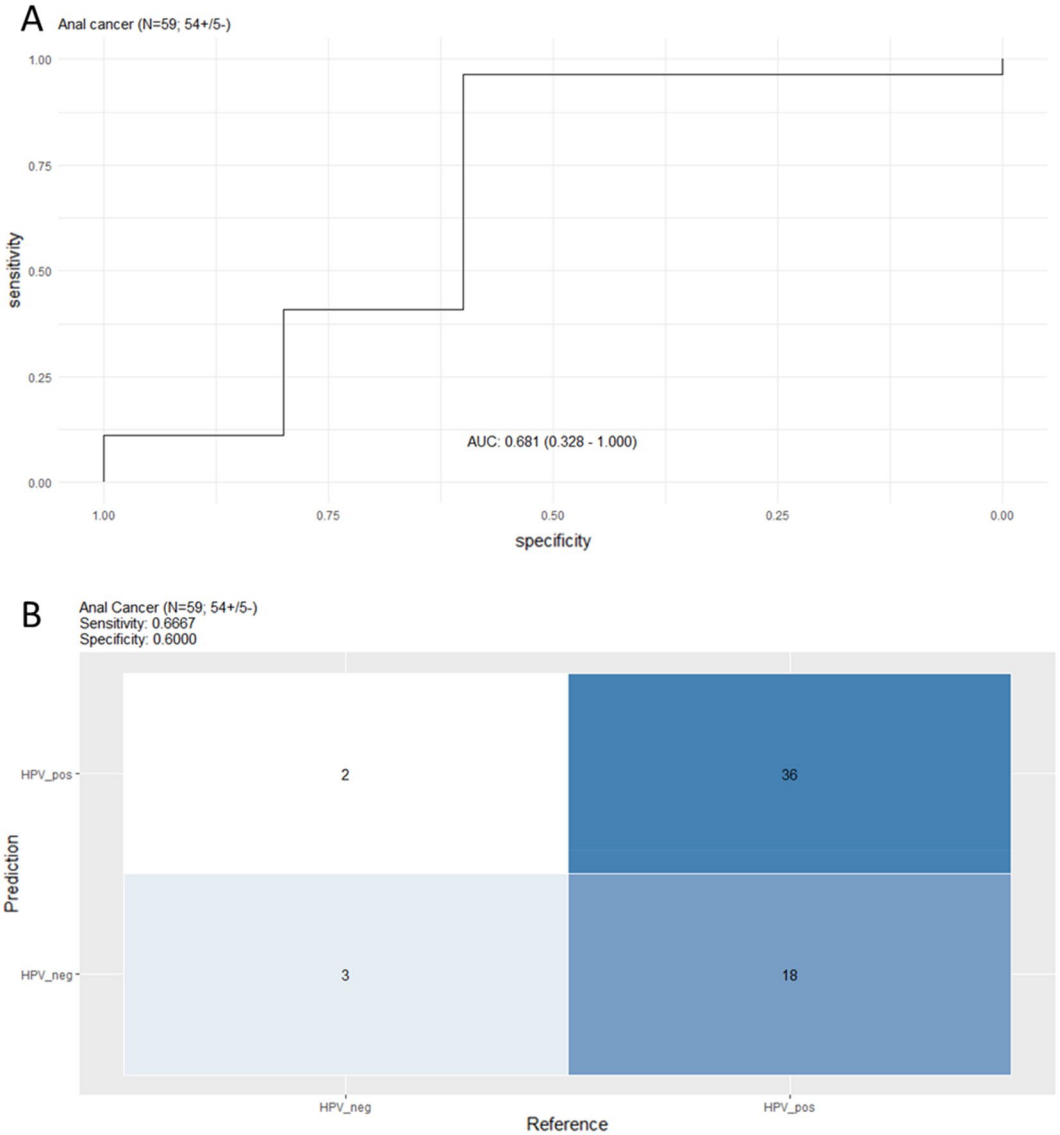
ROC plot (**A**) and confusion matrix (**B**) for the HPV radiomics signature validated on anal cancer patients.

**Figure 3:**
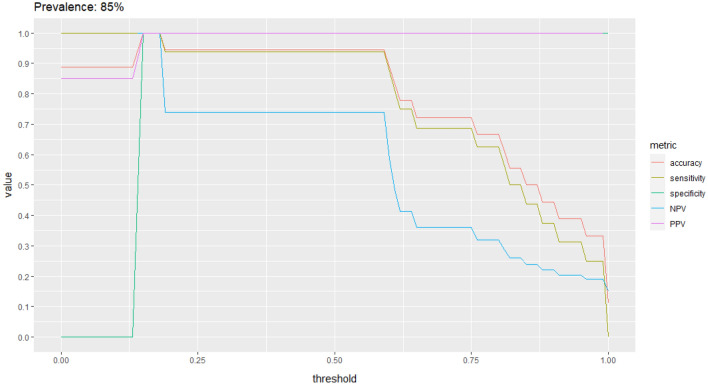
Classification performance plot. The classification performance in the anal cancer dataset, assuming a disease prevalence of 85%, in terms of accuracy (pink line), sensitivity (dark green line), specificity (green line), NPV (blue line) and PPV (purple line) for different decision thresholds.

The model performance on the validation data set of anal cancer patients (n = 59) presents an AUC of 0.681 [95% CI (0.328–1.000)]. The ROC plot and the confusion matrix for the validation set are shown in Fig. 2. Classification performance plot, assuming a disease prevalence of 85%, is reported in Fig. 3.

The signature showed discriminative power also for anal cancer, predicting the probability of p16 + (HPV +), or better the “HPV-ness” of the tumor. Table 1 reports the performance parameters of the external validation in the anal cancer cohort compared to the original validation in OPSSC.

**Table 1:** Performance comparison between original OPSCC validation and anal cancer validation.

	Anal cancer validation	Original OPSCC validation
F1 score	0.782	0.745
Sensitivity	0.666	0.768
Specificity	0.600	0.647
Precision	0.947	0.724
AUC	0.681	0.763

The RQS of the original signature, developed in OPSSC, is 50%. The additional validation with an external dataset, brings the RQS of the signature to 61%. Additionally, the original signature for OPSSC was TRIPOD level 2a (56%) while the new proposed model is a TRIPOD level 4 (57%) signature (See Supplementary Information).

## Discussion

In this study we validated a CT based radiomic signature to predict the p16 status of anal cancer patients. The study provides a proof of concept that molecular information can be inferred from standard medical images by means of radiomics. Previous exploratory radiomic studies that indicated a correlation between HPV infection and heterogeneity of imaging-based tumor density have focused on head and neck cancer^9,17^ and were performed without validation, or only using data from a single institution, for both model development and validation. This is a major problem for the reliability of prediction models based on radiomics signature^7^. Previously published studies report that HPV positive tumors are more homogenous in CT density^18,19^. The homogeneity in turn can represent one of the reasons why HPV + tumors have a better therapeutic outcome and prognosis^20^. We show that the signature which was previously developed and validated in head and neck cancer, also shows discrimination power for anal cancer patients. This suggests that it would be possible to cautiously generalize these findings on tumor radiomics features beyond OPSCC. Furthermore, this study provides an additional insight into p16 (HPV) imaging phenotype. We observed that the p 16 positive tumors are characterized by lower contrast uptake, lower minimum density, and higher changes in the intensity of adjacent voxels. It is worth noting that is not possible to distinguish between p16 + and p16- anal tumors by visual inspection of the CT scan alone, and it has been proven a difficult task also for oropharyngeal cancer^21^. The difference in prevalence of anal cancer is important to consider in order to assess the performance of the signature. The prevalence of HPV positive anal cancers is 85%^15^ and needs to be taken into account in the implementation of the signature as decision support tool for clinicians. The main aim of such signature applied to anal cancer would be the identification of HPV-negative tumors, which can be assessed using the specificity and the NPV (Fig. 2).

Including data from different institutions introduces variability in image acquisition and reconstruction, which affects radiomic features^22^. Besides variability in CT imaging, demographic differences also have to be considered. Developing a model on a single cohort is unlikely to capture the diversity that exists across data from different centers, resulting in a model with poor generalizability, unsuited for routine clinical use. Since the original radiomics signature was developed on a heterogeneous dataset, coming from 5 different institutions, the robustness and the widespread application was greatly improved. The patient cohort for the anal cancer validation was also acquired from two different centers with different scanners and image acquisition parameters (Table S1). Even considering the small number of data available, the model has retained enough discriminating power to correctly classify 70% of the anal cancer patients.

Another open question is related to the rate of false positive in the immunohistochemical test for 16p. Part of the OPSCC patients that test positive for p16 immunohistochemistry are in fact HPV DNA negative^23^. This is also true for anal cancer patients^24^. It is worth noting that HPV + status does not imply per definition p16 + status and vice versa. Prognosis of HPV + /p16 + is therefore not the same as HPV − /p16 + , most likely because in the latter case tumors are not HPV induced. Furthermore, model class predictions (i.e. predicting either HPV positive or HPV negative), were made with a probability cut-off of 0.5, meaning that the costs for false-positives and false-negatives were considered equal. In clinical practice, false positive have a much higher cost in term of patient management and healthcare quality and should be minimized. To achieve a clinically acceptable level of accuracy, further development and validation would be needed, including HPV DNA testing. The radiomics HPV prediction model, while reliable, should not supersede the traditional clinical decision-making approach, based on universally accepted methodology. However, radiomics has the potential to serve as a time-efficient, complementary method for HPV screening, also, for non-oropharyngeal SCCs^25,26^. Radiomics approaches can be used to perform retrospective biomarker studies on HPV status where tissue samples are not available or in countries where HPV testing is not routinely performed. Furthermore, additional improvement in inferring tumor HPV status may be achieved when combining radiomics with clinical features^27^.

## Conclusion

The discriminating power of the radiomics signature for p16 status determination, developed for OPSCC, was also validated for anal cancer patients. These preliminary but encouraging results may pave the road for further generalization of CT image features of HPV related tumors. The use of a larger cohort with p16 and HPV DNA test data, as well as the inclusion of other possible cancer types which shown a correlation with HPV status would be instrumental in this regard.

## Supplementary Information

Supplementary Information.

## Data Availability

The datasets used and/or analyzed during the current study is available from the corresponding author on reasonable request.
