# Supplementary material for: External validation of a radiomic signature to predict p16 (HPV) status from standard CT images of anal cancer patients
Source: Sci Rep. 2023 May 3;13:7198. doi: 10.1038/s41598-023-34162-3 (PMC10156720; doi:10.1038/s41598-023-34162-3)
Supplement: Supplementary file 1 — Supplementary Information. [file 41598_2023_34162_MOESM1_ESM.docx]

**Supplementary Information**

**External validation of a radiomic signature to predict p16 (HPV) status from standard CT images of anal cancer patients**

Ralph T.H. Leijenaar^1*^, Sean Walsh^1*^, Akshayaa Vaidyanathan^1,2^, Lorenzo Aliboni^1^, Victoria Lopez Sanchez^1^, Michelle Leech^3^, Ronan Joyce^4^, Charles Gillham^4^, Frédéric Kridelka^5^, Roland Hustinx^6^, Denis Danthine^6^, Mariaelena Occhipinti^1^, Wim Vos^1^, Julien Guiot^7^, Philippe Lambin^2,8+^ & Pierre Lovinfosse^6+^

* Ralph T.H. Leijenaar & Sean Walsh have equally contributed as first authors.

+ Philippe Lambin & Pierre Lovinfosse have equally contributed as senior authors.

1. Radiomics (Oncoradiomics SA), Liège, Belgium
2. The D-Lab, Department of Precision Medicine, GROW–School for Oncology and Reproduction, Maastricht University, Maastricht, The Netherlands
3. Applied Radiation Therapy, Discipline of Radiation Therapy, Trinity St. James’s Cancer Institute, Trinity College, Dublin, Ireland
4. Department of Radiation Oncology, St. Luke’s Radiation Oncology Network and St James's Hospital, Dublin, Ireland
5. Department of Obstetrics and Gynecology, University Hospital of Liège, Belgium
6. Department of Nuclear Medicine and Oncological Imaging, University Hospital of Liège, Liège, Belgium
7. Department of Pneumology, University Hospital of Liège, Liège, Belgium
8. Department of Radiology and Nuclear Medicine, GROW–School for Oncology and Reproduction, Maastricht University, Maastricht, The Netherlands

**Corresponding Author:**

Pierre Lovinfosse

Department of Nuclear Medicine and Oncological Imaging,

University Hospital of Liège, Liège, Belgium

[pierre.lovinfosse@chuliege.be](mailto:pierre.lovinfosse@chuliege.be)

**Table 1.** Imaging parameters of patients cohorts

|  | HLG (n = 18) | TCD (n = 41) |
| --- | --- | --- |
| Scanner brand | Phillips 92%  Siemens 4 %  GE Medical System 4 % | GE Medical System 100% |
| Reconstruction Kernel | B 88 %  B31f 6 %  Standard 6% | Standard 100% |
| Pixel Spacing | 1.14 (± 0.28) | 1.03 (± 0.11) |
| Slice Thickness | 3.25 (± 1.33) | 2.5 |

**Radiomics Quality Score - RQS**

| **Criteria** | | |  | **Points** |
| --- | --- | --- | --- | --- |
| 1 | *Image protocol quality*  Well-documented image protocols (for example, contrast, slice thickness, energy, etc.) and/or usage of public image protocols allow reproducibility/replicability | | +2 | + 1 (if protocols are well-documented)  + 1 (if public protocol is used) |
| 2 | *Multiple segmentations*  Possible actions are: segmentation by different physicians/algorithms/software, perturbing segmentations by (random) noise, segmentation at different breathing cycles. Analyse feature robustness to segmentation variabilities | | +1 | + 1 |
| 3 | *Phantom study on all scanners*  Detect inter-scanner differences and vendor-dependent features. Analyse feature robustness to these sources of variability | | 0 | + 1 |
| 4 | *Imaging at multiple time points*  Collect images of individuals at additional time points. Analyse feature robustness to temporal variabilities (for example, organ movement, organ expansion/ shrinkage) | | 0 | + 1 |
| 5 | *Feature reduction or adjustment for multiple testing*  Decreases the risk of overfitting. Overfitting is inevitable if the number of features exceeds the number of samples. Consider feature robustness when selecting features | | +3 | - 3 (if neither measure is implemented)  + 3 (if either measure is implemented) |
| 6 | *Multivariable analysis with non radiomics features*  Permits correlating/inferencing between radiomics and non radiomics features | | +1 | + 1 |
| 7 | *Detect and discuss biological correlates*  Demonstration of phenotypic differences (possibly associated with underlying gene–protein expression patterns) deepens understanding of radiomics and biology | | +1 | + 1 |
| 8 | *Cut-off analyses*  Determine risk groups by either the median, a previously published cut-off or report a continuous risk variable. Reduces the risk of reporting overly optimistic results | | +1 | + 1 |
| 9 | *Discrimination statistics*  Report discrimination statistics (for example, Cstatistic, ROC curve, AUC) and their statistical significance (for example, pvalues, confidence intervals). One can also apply resampling method (for example, bootstrapping, cross-validation) | | +2 | + 1 (if a discrimination statistic and its statistical significance are reported)  + 1 (if a resampling method technique is also applied) |
| 10 | *Calibration statistics*  Report calibration statistics (for example, Calibration-inthe-large/slope, calibration plots) and their statistical significance (for example, *P*values, confidence intervals). One can also apply resampling method (for example, bootstrapping, cross-validation) | | +2 | + 1 (if a calibration statistic and its statistical significance are reported)  + 1 (if a resampling method technique is also applied) |
| 11 | *Prospective study registered in a trial database*  Provides the highest level of evidence supporting the clinical validity and usefulness of the radiomics biomarker | | 0 | + 7 (for prospective validation of a radiomics signature in an appropriate trial) |
| 12 | *Validation*  The validation is performed without retraining and without adaptation of the cut-off value, provides crucial information with regard to credible clinical performance | | +5 | - 5 (if validation is missing)  + 2 (if validation is based on a dataset from the same institute)  + 3 (if validation is based on a dataset from another institute)  + 4 (if validation is based on two datasets from two distinct institutes)  + 4 (if the study validates a previously published signature)  + 5 (if validation is based on three or more datasets from distinct institutes) |
| 13 | *Comparison to ‘gold standard’*  Assess the extent to which the model agrees with/is superior to the current ‘gold standard’ method (for example, TNM-staging for survival prediction). This comparison shows the added value of radiomics | | +2 | + 2 |
| 14 | *Potential clinical utility*  Report on the current and potential application of the model in a clinical setting (for example, decision curve analysis). | | 0 | + 2 |
| 15 | *Cost-effectiveness analysis*  Report on the cost-effectiveness of the clinical application | | 0 | + 1 |
| 16 | *Open science and data*  Make code and data publicly available. Open science facilitates knowledge transfer and reproducibility of the study | | 0 | + 1 (if scans are open source)  + 1 (if region of interest segmentations are open source)  + 1 (if code is open source)  + 1 (if radiomics features are calculated on a set of representative ROIs and the calculated features and representative ROIs are open source) |
|  | | Total points (36 = 100%) | | |

**TRIPOD checklist**

| Title and abstract | |  |
| --- | --- | --- |
| 1 | Identify the study as developing and/or validating a multivariable prediction model, the target population, and the outcome to be predicted. | 1 |
| i | The words developing/development, validation/validating, incremental/added value (or synonyms) are reported in the title | Y |
| ii | The words prediction, risk prediction, prediction model, risk models, prognostic models, prognostic indices, risk scores (or synonyms) are reported in the title | Y |
| iii | The target population is reported in the title | Y |
| iv | The outcome to be predicted is reported in the title | Y |
| 2 | Provide a summary of objectives, study design, setting, participants, sample size, predictors, outcome, statistical analysis, results, and conclusions. | 0 |
| i | The objectives are reported in the abstract | Y |
| ii | Sources of data are reported in the abstract *E.g. Prospective cohort, registry data, RCT data.* | Y |
| iii | The setting is reported in the abstract *E.g. Primary care, secondary care, general population, adult care, or paediatric care. The setting should be reported for both the development and validation datasets, if applicable.* | Y |
| iv | A general definition of the study participants is reported in the abstract *E.g. patients with suspicion of certain disease, patients with a specific disease, or general eligibility criteria.* | Y |
| v | The overall sample size is reported in the abstract | Y |
| vi | The number of events (or % outcome together with overall sample size) is reported in the abstract *If a continuous outcome was studied, score Not applicable (NA)*. | Y |
| vii | Predictors included in the final model are reported in the abstract. For validation studies of well-known models, at least the name/acronym of the validated model is reported *Broad descriptions are sufficient, e.g. ‘all information from patient history and physical examination’. Check in the main text whether all predictors of the final model are indeed reported in the abstract.* | N |
| viii | The outcome is reported in the abstract | Y |
| ix | Statistical methods are described in the abstract *For model development, at least the type of statistical model should be reported. For validation studies a quote like “model’s discrimination and calibration was assessed” is considered adequate. If done, methods of updating should be reported.* | N |
| x | Results for model discrimination are reported in the abstract *This should be reported separately for development and validation if a study includes both development and validation.* | Y |
| xi | Results for model calibration are reported in the abstract *This should be reported separately for development and validation if a study includes both development and validation.* | N |
| xii | Conclusions are reported in the abstract *In publications addressing both model development and validation, there is no need for separate conclusions for both; one conclusion is sufficient.* | Y |
| 3a | Explain the medical context (including whether diagnostic or prognostic) and rationale for developing or validating the multivariable prediction model, including references to existing models. | 1 |
| i | The background and rationale are presented | Y |
| ii | Reference to existing models is included (or stated that there are no existing models) | Y |
| 3b | Specify the objectives, including whether the study describes the development or validation of the model or both. | 1 |
| i | It is stated whether the study describes development and/or validation and/or incremental (added) value | Y |
| Methods | |  |
| 4a | Describe the study design or source of data (e.g., randomized trial, cohort, or registry data), separately for the development and validation data sets, if applicable. | 1 |
| i | The study design/source of data is described *E.g. Prospectively designed, existing cohort, existing RCT, registry/medical records, case control, case series. This needs to be explicitly reported; reference to this information in another article alone is insufficient.* | Y |
| 4b | Specify the key study dates, including start of accrual; end of accrual; and, if applicable, end of follow-up. | 1 |
| i | The starting date of accrual is reported | Y |
| ii | The end date of accrual is reported | Y |
| iii | The length of follow-up and prediction horizon/time frame are reported, if applicable *E.g. “Patients were followed from baseline for 10 years“ and “10-year prediction of…”; notably for prognostic studies with long term follow-up. If this is not applicable for an article (i.e. diagnostic study or no follow-up), then score Not applicable (NA).* | Y |
| 5a | Specify key elements of the study setting (e.g., primary care, secondary care, general population) including number and location of centres. | 1 |
| i | The study setting is reported (e.g. primary care, secondary care, general population) *E.g.: ‘surgery for endometrial cancer patients’ is considered to be enough information about the study setting.* | Y |
| ii | The number of centres involved is reported *If the number is not reported explicitly, but can be concluded from the name of the centre/centres, or if clearly a single centre study, score Yes.* | Y |
| iii | The geographical location (at least country) of centres involved is reported *If no geographical location is specified, but the location can be concluded from the name of the centre(s), score Yes.* | Y |
| 5b | Describe eligibility criteria for participants. | 0 |
| i | In-/exclusion criteria are stated *These should explicitly be stated. Reasons for exclusion only described in a patient flow is not sufficient.* | N |
| 5c | Give details of treatments received, if relevant.  *(i.e. notably for prognostic studies with long term follow-up)* | 1 |
| i | Details of any treatments received are described  *This item is notably for prognostic modelling studies and is about treatment at baseline or during follow-up. The ‘if relevant’ judgment of treatment requires clinical knowledge and interpretation.  If you are certain that treatment was not relevant, e.g. in some diagnostic model studies, score Not applicable.* | Y |
| 6a | Clearly define the outcome that is predicted by the prediction model, including how and when assessed. | 1 |
| i | The outcome definition is clearly presented *This should be reported separately for development and validation if a publication includes both.* | Y |
| ii | It is described how outcome was assessed (including all elements of any composite, for example CVD [e.g. MI, HF, stroke]). | R |
| iii | It is described when the outcome was assessed (time point(s) since T0) | R |
| 6b | Report any actions to blind assessment of the outcome to be predicted. | 0 |
| i | Actions to blind assessment of outcome to be predicted are reported *If it is clearly a non-issue (e.g. all-cause mortality or an outcome not requiring interpretation), score Yes. In all other instances, an explicit mention is expected*. | N |
| 7a | Clearly define all predictors used in developing or validating the multivariable prediction model, including how and when they were measured. | 0 |
| i | All predictors are reported *For development, “all predictors” refers to all predictors that potentially could have been included in the ‘final’ model (including those considered in any univariable analyses). For validation, “all predictors” means the predictors in the model being evaluated.* | N |
| ii | Predictor definitions are clearly presented | N |
| iii | It is clearly described how the predictors were measured | N |
| iv | It is clearly described when the predictors were measured | N |
| 7b | Report any actions to blind assessment of predictors for the outcome and other predictors. | 0 |
| i | It is clearly described whether predictor assessments were blinded for outcome *For predictors for which it is clearly a non-issue (e.g. automatic blood pressure measurement, age, sex) and for instances where the predictors were clearly assessed before outcome assessment, score Yes. For all other predictors an explicit mention is expected.* | N |
| ii | It is clearly described whether predictor assessments were blinded for the other predictors | N |
| 8 | Explain how the study size was arrived at. | 0 |
| i | It is explained how the study size was arrived at *Is there any mention of sample size, e.g. whether this was done on statistical grounds or practical/logistical grounds (e.g. an existing study cohort or data set of a RCT was used)?* | N |
| 9 | Describe how missing data were handled (e.g., complete-case analysis, single imputation, multiple imputation) with details of any imputation method. | 0 |
| i | The method for handling missing data (predictors and outcome) is mentioned *E.g. Complete case (explicit mention that individuals with missing values have been excluded), single imputation, multiple imputation, mean/median imputation. If there is no missing data, there should be an explicit mention that there is no missing data for all predictors and outcome. If so, score Yes. If it is unclear whether there is missing data (from e.g. the reported methods or results), score No. If it is clear there is missing data, but the method for handling missing data is unclear, score No.* | N |
| ii | If missing data were imputed, details of the software used are given *When under 9i explicit mentioning of no missing data, complete case analysis or no imputation applied, score Not applicable.* | N |
| iii | If missing data were imputed, a description of which variables were included in the imputation procedure is given *When under 9i explicit mentioning of no missing data, complete case analysis or no imputation applied, score Not applicable.* | N |
| iv | If multiple imputation was used, the number of imputations is reported *When under 9i explicit mentioning of no missing data, complete case analysis or no imputation applied, score Not applicable.* | N |
| 10a | Describe how predictors were handled in the analyses. | Not applicable |
| 10b | Specify type of model, all model-building procedures (including any predictor selection), and method for internal validation. | Not applicable |
| 10c | For validation, describe how the predictions were calculated. | 1 |
| i. | It is described how predictions for individuals (in the validation set) were obtained from the model being validated  *E.g. Using the original reported model coefficients with or without the intercept, and/or using updated or refitted model coefficients, or using a nomogram, spreadsheet or web calculator.* | Y |
| 10d | Specify all measures used to assess model performance and, if relevant, to compare multiple models.  *These should be described in methods section of the paper (item 16 addresses the reporting of the results for model performance).* | 1 |
| i | Measures for model discrimination are described *E.g. C-index / area under the ROC curve.* | Y |
| ii | Measures for model calibration are described *E.g. calibration plot, calibration slope or intercept, calibration table, Hosmer Lemeshow test, O/E ratio*. | Y |
| iii | Other performance measures are described  *E.g. R2, Brier score, predictive values, sensitivity, specificity, AUC difference, decision curve analysis, net reclassification improvement, integrated discrimination improvement, AIC.* | Y |
| 10e | Describe any model updating (e.g., recalibration) arising from the validation, if done. | Not applicable |
| i | A description of model-updating is given *E.g. Intercept recalibration, regression coefficient recalibration, refitting the whole model, adding a new predictor  If updating was done, it should be clear which updating method was applied to score Yes.  If it is not explicitly mentioned that updating was applied in the study, score this item as ‘Not applicable’.* | NA |
| 11 | Provide details on how risk groups were created, if done.  *If risk groups were not created, score this item as Yes.* | Not applicable |
| i | If risk groups were created, risk group boundaries (risk thresholds) are specified  *Score this item separately for development and validation if a study includes both development and validation. If risk groups were not created, score this item as not applicable.* | NA |
| 12 | For validation, identify any differences from the development data in setting, eligibility criteria, outcome and predictors. | 1 |
| i | Differences or similarities in definitions with the development study are described *Mentioning of any differences in all four (setting, eligibility criteria, predictors and outcome) is required to score Yes.  If it is explicitly mentioned that there were no differences in setting, eligibility criteria, predictors and outcomes, score Yes.* | Y |
| Results | |  |
| 13a | Describe the flow of participants through the study, including the number of participants with and without the outcome and, if applicable, a summary of the follow-up time. A diagram may be helpful. | 0 |
| i | The flow of participants is reported | N |
| ii | The number of participants with and without the outcome are reported *If outcomes are continuous, score Not applicable.* | NA |
| iii | A summary of follow-up time is presented *This notably applies to prognosis studies and diagnostic studies with follow-up as diagnostic outcome. If this is not applicable for an article (i.e. diagnostic study or no follow-up), then score Not applicable.* | NA |
| 13b | Describe the characteristics of the participants (basic demographics, clinical features, available predictors), including the number of participants with missing data for predictors and outcome. | 0 |
| i | Basic demographics are reported | N |
| ii | Summary information is provided for all predictors included in the final developed/validated model | N |
| iii | The number of participants with missing data for predictors is reported | N |
| iv | The number of participants with missing data for the outcome is reported | N |
| 13c | For validation, show a comparison with the development data of the distribution of important variables (demographics, predictors and outcome). | 0 |
| i | Demographic characteristics (at least age and gender) of the validation study participants are reported along with those of the original development study | N |
| ii | Distributions of predictors in the model of the validation study participants are reported along with those of the original development study | N |
| iii | Outcomes of the validation study participants are reported along with those of the original development study | N |
| 14a | Specify the number of participants and outcome events in each analysis. | Not applicable |
| 14b | If done, report the unadjusted association between each candidate predictor and outcome. | Not applicable |
| 15a | Present the full prediction model to allow predictions for individuals (i.e., all regression coefficients, and model intercept or baseline survival at a given time point). | Not applicable |
| 15b | Explain how to use the prediction model. | Not applicable |
| 16 | Report performance measures (with confidence intervals) for the prediction model.  *These should be described in results section of the paper (item 10 addresses the reporting of the methods for model performance).* | 1 |
| i | A discrimination measure is presented *E.g. C-index / area under the ROC curve.* | Y |
| ii | The confidence interval (or standard error) of the discrimination measure is presented | Y |
| iii | Measures for model calibration are described *E.g. calibration plot, calibration slope or intercept, calibration table, Hosmer Lemeshow test, O/E ratio.* | Y |
| iv | Other model performance measures are presented *E.g. R2, Brier score, predictive values, sensitivity, specificity, AUC difference, decision curve analysis, net reclassification improvement, integrated discrimination improvement, AIC.* | Y |
| 17 | If done, report the results from any model updating (i.e., model specification, model performance, recalibration). | Not applicable |
| 0 | Model updating was done *If "No", then it is not necessary to answer 17i-17v.* | N |
| i | The updated regression coefficients for each predictor in the model are reported  *If model updating was described as ‘not needed’, score Yes.* | N |
| ii | The updated intercept or cumulative baseline hazard or baseline survival (for at least one time point) is reported  *If model updating was described as ‘not needed’, score Yes.* | N |
| iii | The discrimination of the updated model is reported | N |
| iv | The confidence interval (or standard error) of the discrimination measure of the updated model is reported | N |
| v | The calibration of the updated model is reported | N |
| Discussion | |  |
| 18 | Discuss any limitations of the study (such as nonrepresentative sample, few events per predictor, missing data). | 1 |
| i | Limitations of the study are discussed *Stating any limitation is sufficient.* | Y |
| 19a | For validation, discuss the results with reference to performance in the development data, and any other validation data. | 1 |
| i | Comparison of results to reported performance in development studies and/or other validation studies is given | Y |
| 19b | Give an overall interpretation of the results considering objectives, limitations, results from similar studies and other relevant evidence. | 1 |
| i | An overall interpretation of the results is given | Y |
| 20 | Discuss the potential clinical use of the model and implications for future research. | 0 |
| i | The potential clinical use is discussed  *E.g. an explicit description of the context in which the prediction model is to be used (e.g. to identify high risk groups to help direct treatment, or to triage patients for referral to subsequent care).* | N |
| ii | Implications for future research are discussed *E.g. a description of what the next stage of investigation of the prediction model should be, such as ”We suggest further external validation”.* | N |
| Other information | |  |
| 21 | Provide information about the availability of supplementary resources, such as study protocol, web calculator, and data sets. |  |
| i | Information about supplementary resources is provided | Y |
| 22 | Give the source of funding and the role of the funders for the present study. | 1 |
| i | The source of funding is reported or there is explicit mention that there was no external funding involved | Y |
| ii | The role of funders is reported or there is explicit mention that there was no external funding | Y |
|  |  |  |
|  |  |  |
|  | Number of applicable TRIPOD items | 27 |
|  | Number of TRIPOD items adhered | 16 |
|  | OVERALL adherence to TRIPOD | 59% |
